# Relationship between Tumor Heterogeneity Measured on FDG-PET/CT and Pathological Prognostic Factors in Invasive Breast Cancer

**DOI:** 10.1371/journal.pone.0094017

**Published:** 2014-04-10

**Authors:** Michael Soussan, Fanny Orlhac, Marouane Boubaya, Laurent Zelek, Marianne Ziol, Véronique Eder, Irène Buvat

**Affiliations:** 1 Paris 13 University, Sorbonne Paris Cité, Bobigny, France; 2 Department of Nuclear Medicine, AP-HP, Avicenne University Hospital, Bobigny, France; 3 IMNC - UMR 8165 CNRS - Paris 7 and Paris 11 Universities, Orsay, France; 4 Clinical Research Unit, AP-HP, Avicenne University Hospital, Bobigny, France; 5 Department of Oncology, AP-HP, Avicenne University Hospital, Bobigny, France; 6 Department of Pathology, AP-HP, Jean Verdier Hospital, Bondy, France; 7 CEA-SHFJ, Orsay, France; University of Nebraska Medical Center, United States of America

## Abstract

**Background:**

There is currently little support to understand which pathological factors led to differences in tumor texture as measured from FDG PET/CT images. We studied whether tumor heterogeneity measured using texture analysis in FDG-PET/CT images is correlated with pathological prognostic factors in invasive breast cancer.

**Methods:**

Fifty-four patients with locally advanced breast cancer who had an initial FDG-PET/CT were retrospectively included. In addition to SUVmax, three robust textural indices extracted from 3D matrices: High-Gray-level Run Emphasis (HGRE), Entropy and Homogeneity were studied. Univariate and multivariate logistic regression was used to identify PET parameters associated with poor prognosis pathological factors: hormone receptor negativity, presence of HER-2 and triple negative phenotype. Receiver operating characteristic (ROC) curves and the (AUC) analysis, and reclassification measures, were performed in order to evaluate the performance of combining texture analysis and SUVmax for characterizing breast tumors.

**Results:**

Tumor heterogeneity, measured with HGRE, was higher in negative estrogen receptor (p = 0.039) and negative progesterone receptor tumors (p = 0.036), and in Scarff-Bloom-Richardson grade 3 tumors (p = 0.047). None of the PET indices could identify HER-2 positive tumors. Only SUVmax was positively correlated with Ki-67 (p<0.0004). Triple negative breast cancer (TNBC) exhibited higher SUVmax (Odd Ratio = 1.22, 95%CI [1.06–1.39],p = 0.004), lower Homogeneity (OR = 3.57[0.98–12.5],p = 0.05) and higher HGRE (OR = 8.06[1.88–34.51],p = 0.005) than non-TNBC. Multivariate analysis showed that HGRE remained associated with TNBC (OR = 5.27[1.12–1.38],p = 0.03) after adjustment for SUVmax. Combining SUVmax and HGRE yielded in higher area under the ROC curves (AUC) than SUVmax for identifying TNBC: AUC =  0.83 and 0.77, respectively. Probability of correct classification also increased in 77% (10/13) of TNBC and 71% (29/41) of non-TNBC (p = 0.003), when combining SUVmax and HGRE.

**Conclusions:**

Tumor heterogeneity measured on FDG-PET/CT was higher in invasive breast cancer with poor prognosis pathological factors. Texture analysis might be used, in addition to SUVmax, as a new tool to assess invasive breast cancer aggressiveness.

## Introduction

Tumor texture analysis in FDG PET/CT is a research area of growing interest in the field of oncology and might offer new insights into the characterization of tumors. Texture analysis has recently shown promising results in predicting response to therapy in cervix, head and neck, lung and oesophageal cancer [1], [2]. Texture analysis consists in a variety of mathematical methods describing the relationships between the grey level intensity of voxels and their position within a delineated volume of interest [3]. This method allows for an objective evaluation of how granular or coarse a tumor seems to be at visual analysis. The concept of biological heterogeneity is well known in tumors, and has been recently highlighted by the expression of genomic tumor heterogeneity with important implications for treatment and resistance [4]. Tumor heterogeneity is classically associated with cellular proliferation, necrosis, hypoxia and angiogenesis, all of these factors being related with more tumoral aggressiveness and poorer prognosis in many cancers [5]. Yet, there is currently little support to understand which histological or biological factors led to differences in tumor texture as measured from FDG PET/CT images [3].

FDG PET/CT has proved to be a valuable tool in the staging of locally advanced and inflammatory breast cancer, allowing for the detection of extra-axillary lymph nodes and distant metastases [6]. The hormone receptor negativity, the presence of HER-2 and triple negative phenotype are associated with aggressive histological factors and poor prognosis in breast cancers [7], [8]. In this setting, FDG PET/CT texture analysis might yield new informative data related to metabolic heterogeneity of breast cancer tumors, and as well as add to our understanding of the biologic behavior of this disease.

The purpose of our study was to evaluate whether tumor heterogeneity measured using texture analysis in FDG PET/CT images could be correlated with pathological prognostic factors in invasive breast cancer.

## Methods

### Patient population

This study was approved by the local institutional review board (Ile-de-France X), with waiver of informed consent (data were analyzed anonymously), and was done according to the revised version of the Declaration of Helsinki (2000). Seventy-seven consecutive patients scanned from July 2008 to March 2012 were included in this retrospective study. They all had a large and/or locally advanced and/or inflammatory biopsy-proven breast cancer (T2, T3 or T4) and an initial FDG PET/CT scan before receiving chemotherapy. Eighteen patients were excluded because of delayed acquisition time post-injection. Five patients were excluded because of small tumor volume (<5 mL) leading to uncertain texture analysis. Therefore, the study population included 54 women. Clinical stage was determined according to the American Joint Committee on Cancer (AJCC) 6th edition [9]. Tumor size and T stage were assessed by clinical examination, ultrasound imaging and/or MR imaging.

### Tumor Histology and Immunohistochemistry (IHC) analysis

Tumor type was determined on the core needle biopsy performed before chemotherapy or surgery. Histological grade was determined using the modified Scarff-Bloom-Richardson (SBR) system [10]. Immunohistochemical tests were performed on formalin-fixed, paraffin embedded tissues, using specific antibodies directed against ER (mouse monoclonal, NCL-ER-6F11, Novocastra; dilution 1∶100)**,** PR (mouse monoclonal, NCL-L-PGR-312/2, Novocastra; dilution 1∶200) and c-erbB-2 oncoproteine (polyclonal antibody, DAKO, 1/1000). All immunostainings were performed on a Leica Bond-max automated immunostainer (Leica Microsystems, Newcastle, UK).

Tumors showing moderate or high positivity of at least 10% of cells using ER or PR antibody were classified as ER positive or PR positive, respectively. Tumors were considered to overexpress HER-2 (3+) if more than 30% of invasive tumor cells showed definite membrane staining. Tumors with an IHC score of 2+ were further tested using FISH (fluorescence in situ hybridization). Tumors 2+ with a positive FISH were classified as HER-2 positive. Tumors 2+ with a negative FISH were classified as HER-2 negative. Tumors with an IHC score of 0 or 1+ were considered to be HER-2 negative. A Ki-67 index (percentage of Ki-67-positive cancer nuclei) was also determined (MIB-1, DAKO, dilution 1/50). TNBC were defined as hormone receptor-negative and HER-2-negative tumors.

### PET/CT protocol

PET/CT acquisitions were performed 79+/-9 [range: 59–90] minutes following intravenous injection of 3 MBq/kg of FDG. Serum glucose level was <1.4 g/L at the time of injection for all patients. All FDG-PET/CT images were acquired using a Gemini TF PET/CT scanner (Philips Medical Systems, Netherlands). The Gemini TF is a TOF-capable, fully 3-dimensional PET scanner together with a 16-slice Brilliance CT scanner. CT images were obtained without contrast media injection using the following settings: 120 KV, 100 mA, collimation 16×1.5 mm, pitch of 0.69, slice thickness of 3 mm and increment of 1.5 mm. PET images were reconstructed using a BLOB-OS-TF list-mode iterative algorithm with 2 iterations and 33 subsets. A single scatter-simulation model was used for scatter correction [11] and attenuation correction was performed based on the CT. No post-reconstruction smoothing filter was used. The image voxel size was 4 mm×4 mm×4 mm for PET and 1.17 mm×1.17 mm×1.5 mm for CT. SUVs were calculated from the reconstructed activity concentration values and normalized to body weight.

### Tumor analysis

A 3D solid box was first loosely drawn around each breast tumor so as not to include surrounding regions with high activity. The tumor was then automatically delineated using the approach initially described by Nestle et al [12] where the threshold was defined by: T = β*I70+Ibgd with β = 0.3. The β parameter was optimized using 3 acquisitions of a Jaszczak phantom including spheres from 0.98 to 3.12 cm in diameter, with sphere to background activity ratios varying from 2.96 to 10 [13]. I70 was the mean uptake in a contour containing all voxels with a value greater than 70% of the maximum uptake in the tumor. Ibgd was defined as the mean uptake in a shell of 2 voxels thickness located at 6 voxels from the region used to calculate I70 and only voxels with uptake less than 2.5 SUV units were included in the calculation of Ibgd. The mean SUV (SUVmean) was then calculated in the resulting tumor volume (MV).

### Texture analysis

All textural indices were calculated from the delineated tumor volume as defined above. Voxel values within the segmented tumors were first resampled to yield a finite range of 64 discrete values between the minimum and maximum SUV in the tumor, using:
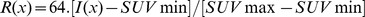
where I(x) is the SUV of voxel x in the original image, SUVmin and SUVmax are the minimum and maximum SUV in the VOI, and R(x) is the resampled value of voxel x. The role of such resampling is to reduce noise and normalize uptake across patients.

Two 3D matrices describing texture heterogeneity were calculated from the delineated tumors. The co-occurence matrix (CM), describing pair wise arrangement of voxels is a 3D matrix related to texture heterogeneity at a local level [14]. The gray-level run length matrix (GLRLM), describing the alignment of voxels with the same intensity is a 3D matrix related to texture heterogeneity at a regional level [15].

In this study, we focused on 3 textural indices among 31 that were initially calculated: Homogeneity and entropy calculated from the CM, and High-Gray-level Run Emphasis (HGRE) calculated from the GLRLM, all as defined in Haralick et al. [14] and Amadasun et al. [16]. Homogeneity measures the local homogeneity of a pixel pair: the homogeneity is expected to be large if the gray levels of each pixel pair are similar. Entropy measures the randomness of a gray-level distribution: the entropy is expected to be high if the gray levels are distributed randomly throughout the tumor region. HGRE measures the distribution of segments of high intensity (high levels of gray). The value is expected to be large if the number of segments of high intensity is high.

These three textural indices were selected as they were found to be robust with respect to the tumor delineation method and were not highly correlated one with another [17]. In summary, 5 PET-derived indices were calculated for each patient: Homogeneity, Entropy, HGRE, SUVmax, and SUVmean.

### Statistical analysis

Univariate logistic regression was used to identify the association between TNBC and all PET indices. Factors that were not linear in the logit were dichotomized at the median. Then, multivariate analysis was performed including factors with p<0.05 in the univariate analysis. Because of strong correlation between texture parameters, separate models were used. The discrimination of scores was assessed using univariate and multivariate receiver operating characteristic (ROC) curves and the areas under the ROC curves (AUC) [18] were compared using a DeLong's test [19]. Because reclassification measures can offer incremental information over the AUC [20], the net reclassification improvement (NRI) was measured when combining SUVmax and textural indices. The net reclassification improvement (NRI) measure is used to assess whether adding texture analysis to SUVmax results in a better identification of patients with TNBC [20]. Any increase in probability of having a TNBC in patients with TNBC implies improved classification, and any decrease in probability indicates worse reclassification. The improvement in reclassification can be quantified by the NRI. The NRI is the sum of the net difference between the proportion of patients correctly reclassified and the proportion of patients incorrectly reclassified (ideal value is 2) [20]. Correlation between level of Ki-67 on biopsy sample and PET indices was tested using Pearson's coefficient correlation. All tests were two-sided at a 0.05 significance level. Analyses were performed using R statistical software version 2.15.2 (The R Foundation for Statistical Computing, Vienna, Austria).

## Results

### Patients and tumor characteristics

FDG PET/CT scans were performed in 54 patients (median age 57, range 31–84 years) with invasive breast carcinoma. Tumor characteristics are listed in Table 1. The median tumor size was 39 mm (range 20–120). 25 patients had a clinical stage II according to the AJCC 6th edition, 15 had a stage III while 14 had a stage IV. Invasive ductal carcinoma was diagnosed in the majority of patients (49/54, 91%). Three patients had an invasive lobular carcinoma (3/54, 5%). The SBR grade was 3 in 76% of patients (41/54). ER and PR were positive in 78% (38/54) and 35% (19/54) of tumors, respectively. Overexpression of HER-2 was found in 24% (13/54) of tumors. 24% of patients (13/54) had a TNBC.

**Table 1:** Patient and breast carcinoma characteristics (n = 54).

**Clinical Stage**	
***IIA***	17 (31)
***IIB***	8 (15)
***III***	15 (28)
***IV***	14 (26)
**Tumor size (mm)**	39 (20–120)
**Histology**	
***Invasive ductal carcinoma***	49 (91)
***Invasive lobular carcinoma***	3 (5)
***Others***	2 (4)
**Histological grade**	
***1/2***	13 (24)
***3***	41 (76)
**ER status**	
***Negative***	16 (30)
***Positive***	38 (70)
**PR status**	
***Negative***	35 (65)
***Positive***	19 (35)
**HER-2**	
***Negative***	41 (76)
***Positive***	13 (24)
**Triple negative breast cancer (TNBC)**	
***TNBC***	13 (24)
***Non-TNBC***	41 (76)

Data are presented as number (frequency) or median (range). ER: Estrogen Receptor; PR: Progesterone Receptor.

### PET texture analysis versus ER, PR, HER-2 status and Ki-67

Tumor heterogeneity assessed by textural indices homogeneity and HGRE was significantly different according to pathological analysis (Table 2 and Table S1). HGRE was higher in negative ER tumors than in positive ER tumors (Odd Ratio OR [95% CI] = 3.57 [1.06;12.5], p = 0.039), negative PR tumors compared to positive PR tumors (OR = 4 [1.09;14.28], p = 0.036) and SBR grade 3 tumors compared to non-grade 3 tumors (OR = 5.22 [1.02;26.72], p = 0.047). Homogeneity was lower in TN phenotype tumors. SUVmax and SUVmean, were also higher in case of negative ER and SBR grade 3 tumors.

**Table 2:** Textural and SUV indices as a function of pathological characteristics of breast cancer at univariate logistic regression.

	Homogeneity (<0.15)		Entropy (>2.2)		HGRE (>850)		SUVmax		SUVmean	
	OR [95% CI]	p	OR [95% CI]	p	OR [95% CI]	p	OR [95% CI]	p	OR [95% CI]	p
**ER negativity**	3.22 [0.94–11.05]	0.06	0.57 [0.13–2.39]	0.44	3.57 [1.06–12.5]	0.039*	1.19 [1.05–1.35]	0.005*	1.40 [1.12–1.79]	0.004*
**PR negativity**	1.46 [0.42–5.03]	0.55	2.33 [0.67–8.33]	0.18	4 [1.09–14.28]	0.036*	1.08 [0.98–1.20]	0.11	1.20 [0.98–1.47]	0.07
**HER-2 positivity**	1.51 [0.51–5.56]	0.54	0.45 [0.12–1.72]	0.24	0.57 [0.15–2.15]	0.4	0.96 [0.86–1.07]	0.48	0.93 [0.76–1.15]	0.51
**High grade (SBR 3)**	3.20 [0.6–16.7]	0.18	1.29 [0.32–5.12]	0.72	5.22 [1.02–26.72]	0.047*	1.13 [0.99–1.27]	0.060	1.32 [1.02–1.7]	0.033
**TN phenotype**	3.57 [0.98–12.5]	0.05	2.28 [0.44–11.85]	0.33	8.06 [1.88–34.51]	0.005*	1.22 [1.06–1.39]	0.005*	1.51 [1.15–1.98]	0.003*

*: p<0.05.

OR: Odd Ratio

95% CI: 95% confidence.

SBR: Scarff-Bloom-Richardson.

ER: Estrogen Receptor;

PR: Progesterone Receptor.

HGRE: High-Gray-level Run Emphasis.

Factors that were not linear in the logit were dichotomized at the median.

Entropy was not associated with any of the histological features.

None of the PET indices could identify tumors with overexpression of HER-2.

SUV indices were significantly positively correlated with Ki-67 with r ranging from 0.50 to 0.54 (p<0.0004), unlike textural indices.

Homogeneity, Entropy and HGRE were not linearly correlated with SUV max (Pearson correlation r = −0.05, 0.15, and 0.29). HGRE was not linearly correlated with MV (r = −0.13), whereas Homogeneity and Entropy were moderately correlated with MV (r = 0.76 and 0.58, respectively).

### PET texture analysis for characterizing triple negative breast cancer (TNBC)

Using logistic regression, factors associated with TN phenotype were homogeneity, HGRE and SUVs (Table 2). MV was not associated with TN phenotype (OR = 0.82 [0.23–2.85], p = 0.75).

**Figure 1:**
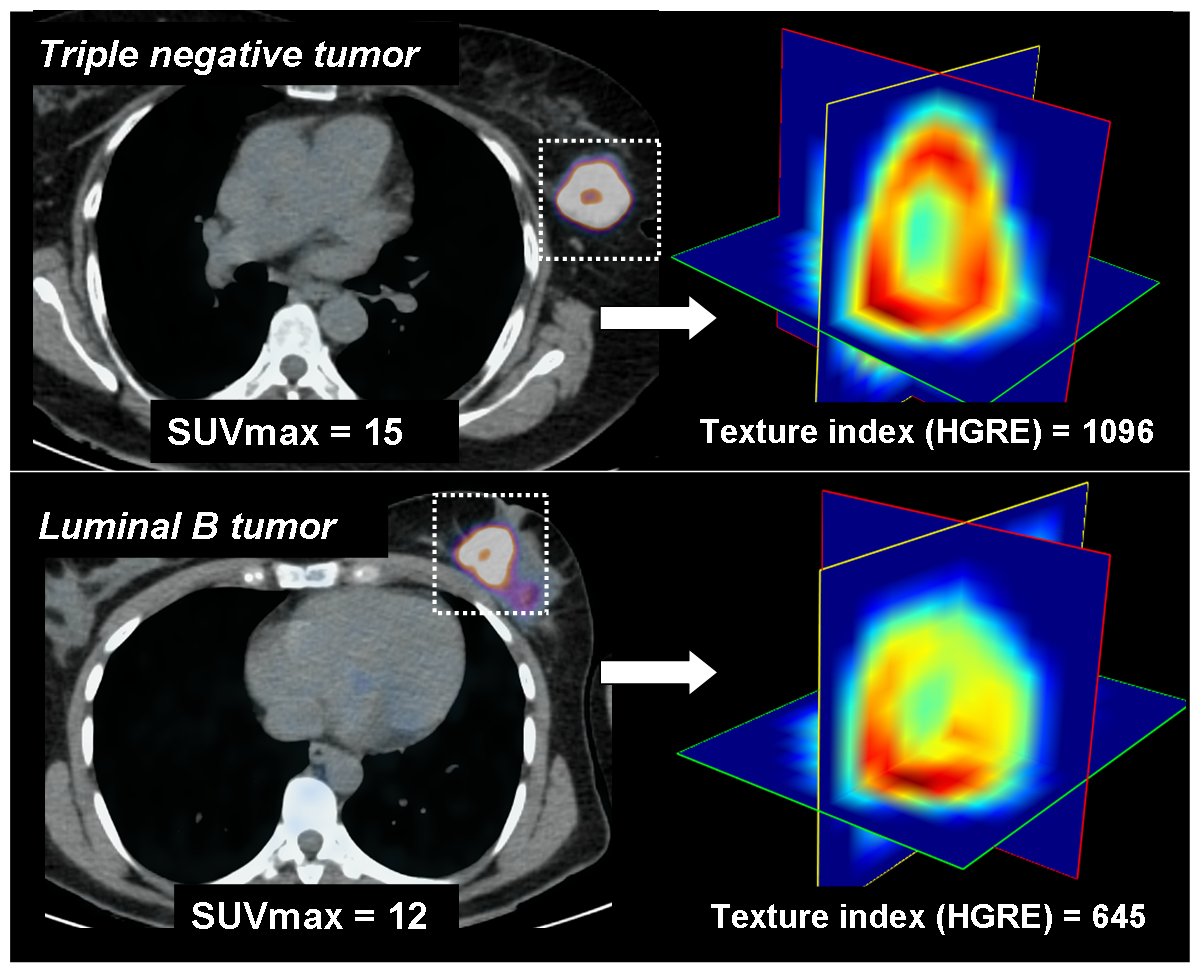
Illustration of tumor heterogeneity at FDG-PET/CT in the different subtypes of breast. Axial PET fusion images (left) and 3D-view of 3 orthogonal plans from the tumor volume (right) after voxel resampling are displayed. Two histologic types of breast tumor are displayed: triple negative (top) and luminal B (bottom) tumors. Both tumors exhibit intense FDG uptake with a central hypometabolic area. The triple negative breast tumor exhibits higher SUVmax and higher textural heterogeneity than the luminal B tumor (right). This example illustrates higher FDG uptake and higher texture heterogeneity in TNBC compared to non-TNBC.

**Figure 2:**
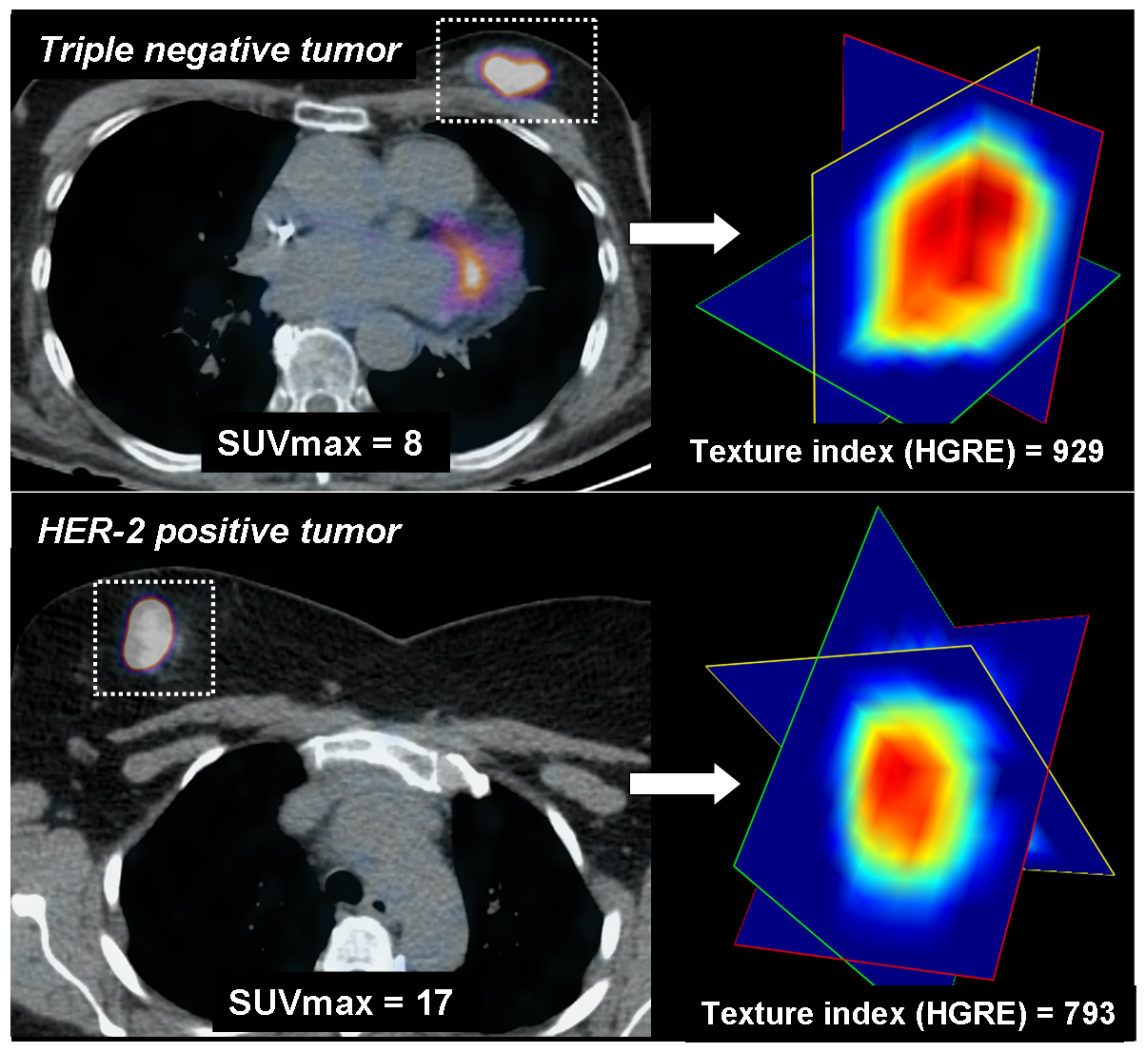
Axial PET fusion images (left) and 3D-view of 3 orthogonal plans from the tumor volume (right) after voxel resampling are displayed. The triple negative breast tumor (top) exhibits lower SUVmax but higher textural heterogeneity than the HER-2 positive breast tumor (bottom). This example illustrates the ability of the HGRE textural index to identify higher heterogeneity despite lower FDG uptake in triple negative breast tumors compared to the non-triple negative breast tumors.

TNBC exhibited more tumor heterogeneity than non-TNBC: lower homogeneity (OR = 3.57 [0.98–12.5], p = 0.05) and higher HGRE (OR = 8.06 [1.88–34.51]) p = 0.005). As expected, SUVmax was also higher in TNBC (OR = 1.22 [1.06–1.39], p = 0.005). Using multivariate logistic regression, HGRE remained associated with TNBC after adjusting the effect of SUVmax (OR = 5.28, p = 0.03), unlike Homogeneity. An illustration of textural heterogeneity is shown in Figures 1 and 2.

**Figure 3:**
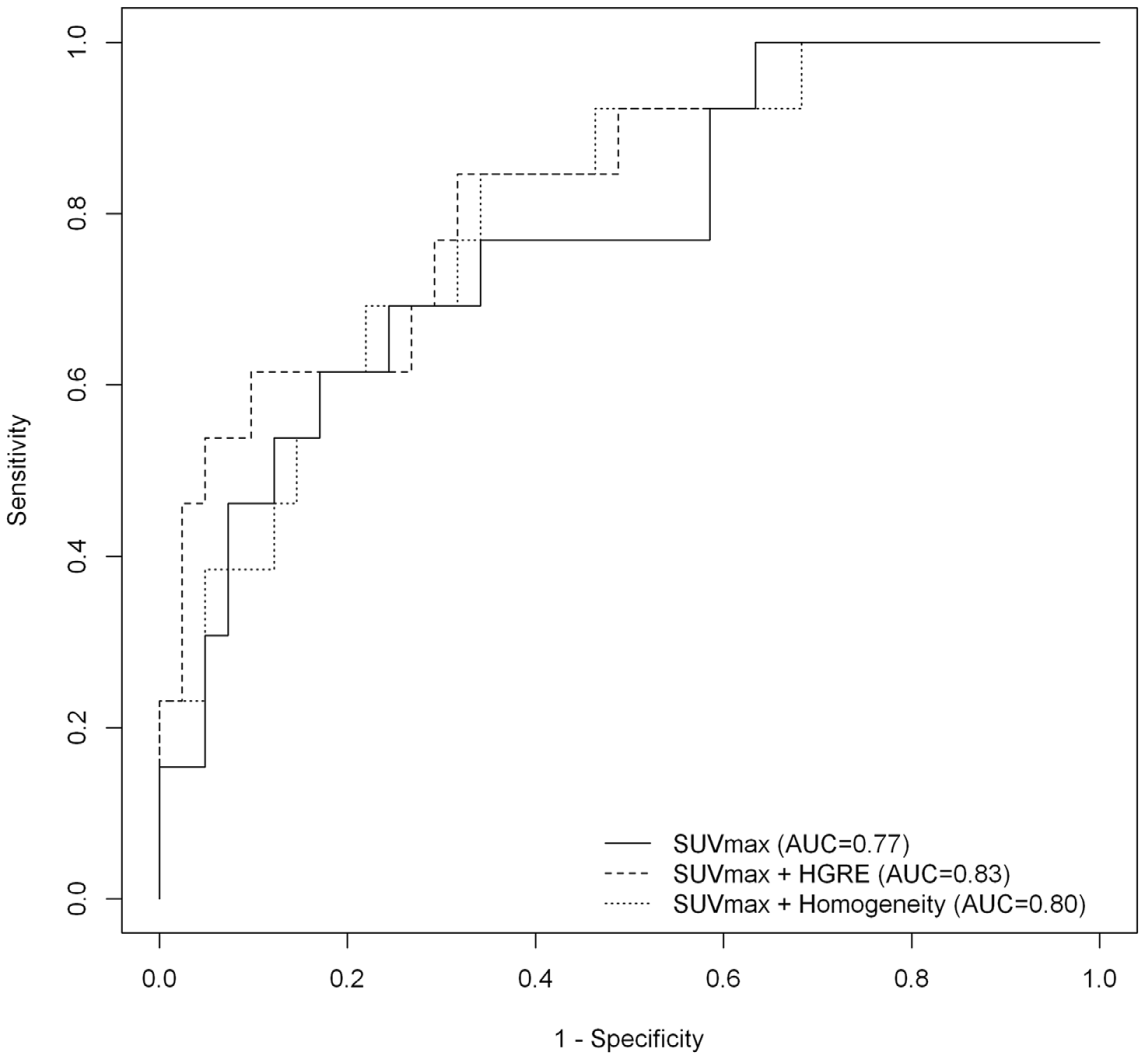
ROC curves analysis. AUC of SUVmax, SUVmax associated with homogeneity and SUVmax with HGRE for identifying TNBC.

**Figure 4:**
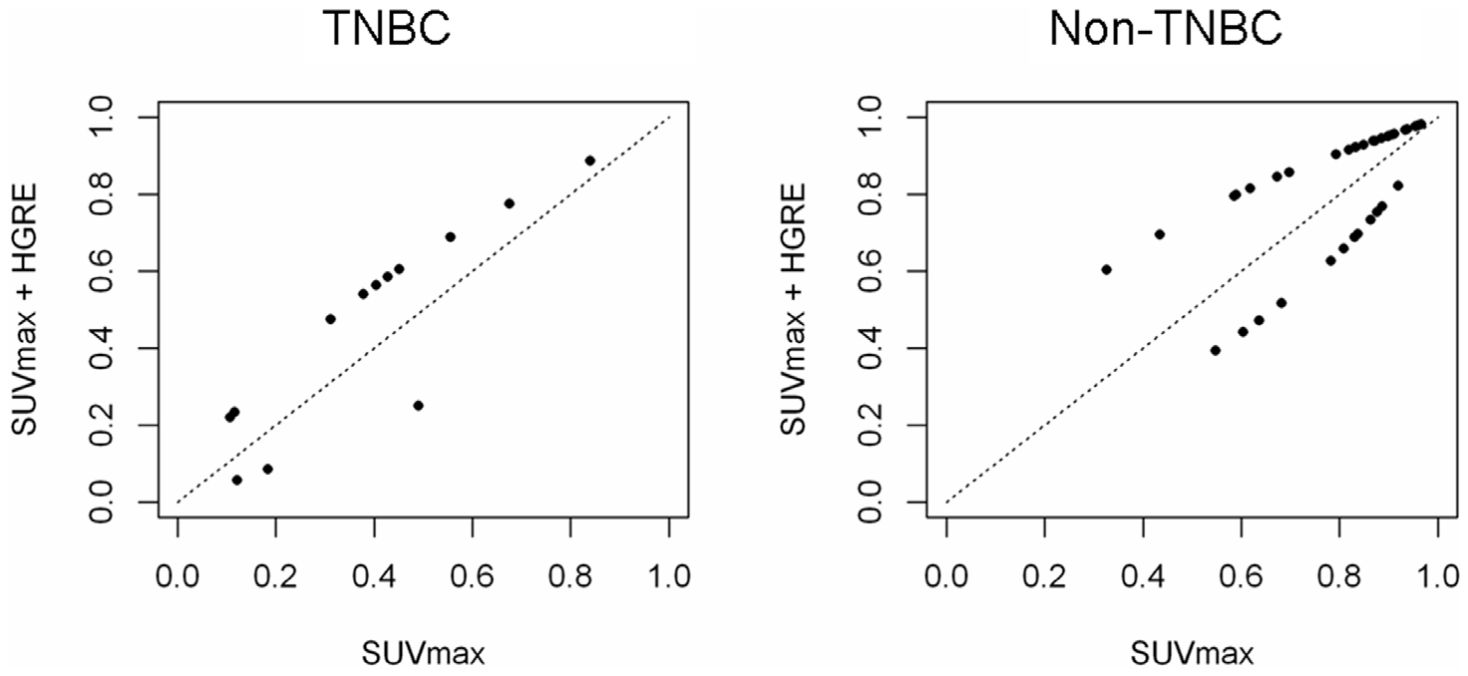
Plots of the patients with TNBC (left) and non-TNBC (right) illustrating the effect of including a texture parameter (HGRE) in addition to SUVmax for classification of breast cancer. In both tumor groups (TNBC and non-TNBC), adding HGRE to SUVmax improves the classification of the tumor if the patient is located above the diagonal. The probability of correct classification increased in 77% of TNBC (10/13) and in 71% (29/41) of non-TNBC (NRI = 0.95, p = 0.003). A perfect combination of indices would reclassify 100% of patients above the diagonal (NRI equal to 2).

In order to evaluate the performance of combining texture analysis and SUVmax for characterizing TN tumors, ROC analysis was performed. AUC was the highest when combining SUVmax and HGRE (AUC = 0.83, Figure 3), without however reaching statistically significant differences compared to SUVmax alone (AUC = 0.77, p = 0.27). Using reclassification method, when combining SUVmax and HGRE, the probability of correct classification increased in 77% (10/13) of TNBC and 71% (29/41) of non-TNBC, leading to an NRI of 0.95 (p = 0.003) (Figure 4).

## Discussion

In this study, we showed that tumor heterogeneity in FDG PET/CT is higher in invasive breast tumors with poor prognosis pathological factors, such as negativity of ER/PR, SBR grade 3 and TN phenotype. Using multivariate analysis, SUVmax and one textural index, High-Gray-level Run Emphasis (HGRE), were both found to be independently associated with TN phenotype. Our results suggest also that the combination of uptake intensity and textural analysis improved the identification of poor prognosis subtype such as TNBC.

Given that texture analysis of breast tumor on PET is a simple and reproducible method and that PET has proved to be a valuable tool in the staging of locally advanced breast tumor, these findings have potential prognostic implications for these patients. Texture analysis could be used in addition to SUVmax, as a new tool to characterize breast tumor aggressiveness.

There is some recent evidence that PET texture analysis can provide significant prognosis data in solid tumors [1], [3]. However, a great variety of indices have been studied and prognostic significance of different texture indices may varies according to the type of solid tumor [3]. So, there is a need to increase the understanding of the histological and biological basis of PET textural indices. Our study is the first assessing the correlation between PET texture analysis with pathological analysis. Interestingly, our data are in line with the concept that tumor heterogeneity is higher in tumor with aggressive pathological factors. HGRE is the most statistically significant textural index associated with poor prognosis pathological analysis in this study. HGRE describes the distribution of segments of high intensity within the tumor and its value is expected to be large if the number of segments of high intensity is high. Using simulated tumors, we showed that the more heterogeneous the tumor uptake, the higher HGRE (results not shown). This direction of variation of textural indices has indeed been observed in our population study. It is well established that tumor exhibits histological heterogeneity because of high proliferative tissue mixed with low proliferative tissue, necrosis and hypoxic areas. These different types of tumoral tissue behave differently regarding the glucose metabolism. Our results suggest that texture analysis could capture the distribution of FDG uptake within a tumor.

The relationship between tumor heterogeneity and pathological analysis in breast cancer has already been investigated in a few MRI studies [21], [22], [23]. Bhooshan et al. showed that textural indices measured in contrast-enhanced MRI could distinguish in situ ductal carcinoma versus invasive ductal carcinoma, as well as invasive ductal carcinoma with positive lymph node [22]. Ahmed et al. recently observed textural differences between TNBC and other types in contrast-enhanced MRI [23]. Similarly, Uematsu et al. showed that very high tumoral signal intensity in T2-weighted MR images, demonstrating tumoral necrosis, was significantly associated with TNBC [24]. The presence of more frequent necrosis in TNBC could partly explain the higher texture heterogeneity observed in FDG PET in these tumors. Necrosis has been shown to be a prognostic factor in invasive breast cancer, associated with early systemic metastasis and accelerated clinical course [25]. Results of Leek et al. also suggest that aggressive tumors rapidly outgrow their vascular supply in certain areas, leading to areas of prolonged hypoxia within the tumor and to subsequent necrosis [26]. Therefore, textural indices derived from PET images might bring an additional insight into tumor biological aggressiveness.

Our results confirmed the previous data regarding the absence of significant influence of HER-2 overexpression on FDG uptake [27], possibly explaining the absence of association between textural features and HER-2 status. The absence of relationship between HER-2 overexpression and FDG uptake is difficult to interpret as it has been demonstrated that HER-2 promotes glycolysis in human breast cancer cells [28].

Previous studies have shown that FDG uptake was higher in breast tumors with poor prognostic pathological factors such as high grade, hormone receptor negativity or TNBC phenotype [27], [29]. Our results confirm these findings and show that proliferative index Ki 67 was correlated only with SUVmax and not with textural indices, suggesting that the textural indices bring a different piece of metabolic information compared to SUVmax. This result highlights the histological complexity of tumors. The proliferative index describes a very small region of the whole tumor, similar to SUVmax corresponding to the highest FDG uptake in a voxel. Textural indices are supposed to describe the spatial distribution of uptake within the tumoral volume, hence the lack of correlation between textural indices and Ki-67.

As a limitation, only tumors with metabolic volume >5 mL were included because texture analysis cannot be reliably performed in small lesions due to the too small number of voxels included in the texture analysis. As a result, unlike SUVmax, texture analysis might not be practical in small primitive lesions or small nodal metastasis. One other limitation is the variability of the scan start time (range 59–90 min) which can lead to different level of tumor to background ratio in the reconstructed image, that could bias texture analysis.

As shown in a recent study where authors demonstrated that there is a positive relationship between the percentage of high washout on dynamic-contrast-enhanced MR and SUVmax [30], correlations between textural indices in FDG PET/CT with MR imaging features of breast tumors could be of interest but are beyond the scope of this study.

In conclusion, tumor heterogeneity measured through textural indices in FDG PET/CT is higher in breast cancer with poor prognosis pathological factors. Texture analysis might be used, in addition to SUVmax, as a new tool to assess invasive breast cancer aggressiveness.

## Supporting Information

Table S1 **Characteristics of all 54 patients with their respective tumor type, HGRE and SUVmax values.**
(DOC)
