# Supplementary material for: Relationship between Tumor Heterogeneity Measured on FDG-PET/CT and Pathological Prognostic Factors in Invasive Breast Cancer
Source: PLoS One. 2014 Apr 10;9(4):e94017. doi: 10.1371/journal.pone.0094017 (PMC3983104; doi:10.1371/journal.pone.0094017)
Supplement: Table S1 — Characteristics of all 54 patients with their respective tumor type, HGRE and SUVmax values. (DOC) [file pone.0094017.s001.doc]

**Table S1.**

| **Patient n°** | **ER** | **PR** | **TNBC** | **HGRE** | **SUVmax** |
| --- | --- | --- | --- | --- | --- |
| 1 | + | + | No | 661.7 | 3.0 |
| 2 | + | + | No | 1058.4 | 8.7 |
| 3 | + | - | No | 1192.1 | 6.8 |
| 4 | + | - | No | 931.0 | 9.8 |
| 5 | + | + | No | 454.8 | 9.4 |
| 6 | + | - | No | 828.5 | 8.0 |
| 7 | + | + | No | 847.9 | 3.6 |
| 8 | + | - | No | 752.8 | 11.5 |
| 9 | + | - | No | 950.6 | 9.2 |
| 10 | + | + | No | 1143.8 | 15.3 |
| 11 | + | - | No | 677.4 | 10.9 |
| 12 | + | - | No | 704.0 | 8.0 |
| 13 | + | - | No | 756.4 | 2.6 |
| 14 | + | - | No | 1104.5 | 12.6 |
| 15 | + | - | No | 787.9 | 20.5 |
| 16 | + | - | No | 624.1 | 7.5 |
| 17 | - | - | Yes | 853.2 | 18.2 |
| 18 | - | - | No | 507.5 | 8.7 |
| 19 | - | - | Yes | 867.9 | 16.7 |
| 20 | + | + | No | 646.0 | 12.3 |
| 21 | + | + | No | 378.7 | 5.6 |
| 22 | + | - | No | 864.2 | 11.0 |
| 23 | + | + | No | 676.7 | 7.3 |
| 24 | - | - | Yes | 896.0 | 17.7 |
| 25 | - | - | Yes | 1075.1 | 22.9 |
| 26 | - | - | Yes | 767.6 | 19.0 |
| 27 | + | - | No | 793.8 | 9.5 |
| 28 | + | + | No | 778.4 | 3.1 |
| 29 | + | - | No | 818.0 | 3.0 |
| 30 | - | - | Yes | 1050.9 | 17.2 |
| 31 | + | - | No | 635.1 | 22.9 |
| 32 | - | - | Yes | 1133.3 | 27.7 |
| 33 | + | + | No | 864.1 | 18.2 |
| 34 | - | - | Yes | 788.1 | 9.0 |
| 35 | - | - | Yes | 514.0 | 11.6 |
| 36 | + | - | No | 949.5 | 11.8 |
| 37 | + | + | No | 535.7 | 16.7 |
| 38 | + | - | No | 1200.7 | 10.8 |
| 39 | - | - | Yes | 859.1 | 20.3 |
| 40 | - | - | Yes | 929.5 | 8.3 |
| 41 | - | + | No | 796.9 | 11.0 |
| 42 | + | + | No | 625.5 | 1.9 |
| 43 | + | - | No | 678.4 | 10.4 |
| 44 | - | - | Yes | 1050.5 | 8.8 |
| 45 | + | - | No | 1077.5 | 17.0 |
| 46 | - | - | No | 815.5 | 17.3 |
| 47 | + | + | No | 793.8 | 17.4 |
| 48 | + | + | No | 716.5 | 14.9 |
| 49 | + | - | No | 753.8 | 5.3 |
| 50 | - | - | Yes | 1095.7 | 15.1 |
| 51 | + | + | No | 712.0 | 3.4 |
| 52 | + | + | No | 816.2 | 7.5 |
| 53 | + | + | No | 1050.1 | 16.3 |
| 54 | + | + | No | 623.4 | 15.5 |

ER: Estrogen Receptor; PR: Progesterone Receptor. TNBC: Triple Negative Breast Cancer; HGRE: High-Gray-level Run Emphasis.
